# Mechanisms of vortices termination in the cardiac muscle

**DOI:** 10.1098/rsos.170024

**Published:** 2017-03-15

**Authors:** D. Hornung, V. N. Biktashev, N. F. Otani, T. K. Shajahan, T. Baig, S. Berg, S. Han, V. I. Krinsky, S. Luther

**Affiliations:** 1 Max Planck Institute DS, BMPG, Gottingen, Germany; 2 University of Exeter, Exeter, UK; 3 Rochester Institute of Technology, Rochester, NY, USA; 4 National Institute of Technology Karnataka, Bangalore, India; 5 Institute for Nonlinear Dynamics, Georg-August-Universität Göttingen, Am Faßberg 17, 37077 Göttingen; 6 INLN, CNRS, Valbonne, France; 7 Department of Pharmacology, University Medical Centre Göttingen, Robert-Koch-Str. 40, 37075 Göttingen, Germany

**Keywords:** nonlinear waves, weakly pinned vortices, cardiac fibrillation, implantable defibrillator

## Abstract

We propose a solution to a long-standing problem: how to terminate multiple vortices in the heart, when the locations of their cores and their critical time windows are unknown. We scan the phases of all pinned vortices in parallel with electric field pulses (E-pulses). We specify a condition on pacing parameters that guarantees termination of one vortex. For more than one vortex with significantly different frequencies, the success of scanning depends on chance, and all vortices are terminated with a success rate of less than one. We found that a similar mechanism terminates also a free (not pinned) vortex. A series of about 500 experiments with termination of ventricular fibrillation by E-pulses in pig isolated hearts is evidence that pinned vortices, hidden from direct observation, are significant in fibrillation. These results form a physical basis needed for the creation of new effective low energy defibrillation methods based on the termination of vortices underlying fibrillation.

## 1. Background

Vortices play a crucial role in many domains of physics, including catalytic waves, and condensed matter physics. In superconductors, the motion of free vortices induces dissipation, so pinning is required to maintain the superconductor state [1]. Pinning and depinning transitions are essential features of superfluid dynamics [2].

Rotating electrical waves (vortices) and their instabilities underlie cardiac chaos (fibrillation) [3–5]. Physics of the vortices is well understood, e.g. [6–10]. But the contemporary method of terminating life-threatening cardiac fibrillation is still aimed at termination of not vortices, but all waves in the heart [11,12]. It delivers a high-energy electric shock, which is damaging and painful. Research aimed at reducing the energy to a non-damaging, pain-free level gave rise to methods [13–18] aimed at terminating vortices rather than all waves. We investigate mechanisms of vortices termination by electric field pulses (E-pulses).

Over a century ago, it was found that a single vortex (rotating wave or anatomical reentry) in a heart can be terminated with an electric pulse [19]. An electrode was placed close to the anatomical obstacle around which the wave rotates and a small energy electric pulse was delivered within a certain time interval, called the critical window, or vulnerable window (VW) (note that for a rotating wave, such intervals repeat within each lap).

This approach alone cannot terminate fibrillation because there are multiple rotating waves with unknown and changing geometric locations and phases [5]. That is, we have two main problems: (i) the geometric positions of their cores, and (ii) the positions of their critical time windows, are not known during fibrillation. An approach to overcome problem (i) has been previously developed [13,14]. Owing to the bi-domain electric nature of cardiac muscle [20,21], every defect in it that can serve as a pinning centre for a vortex, is at the same time an electric inhomogeneity. This allows an E-pulse to excite the cores of all pinned vortices simultaneously, regardless of the geometric positions of their cores.

Approaches to resolve problem (ii) are being developed. They are aimed at delivering a pulse into VWs of all vortices without knowing their relative phases (by phases we mean the phases of oscillations, i.e. ‘positions in time’). One of them is the phase scanning by E-pulses, with a phase step that is shorter than the VW, for all vortices in parallel. It was tested in experiment to terminate a vortex in a rabbit heart preparation [22]. Scanning with periodic E-pulses was used to terminate fibrillation [15,16]. Termination of one vortex with periodic E-pulses was numerically investigated in [23–25].

## 2. Theory

### 2.1 Pinned vortices

In this paper, we investigate termination of multiple vortices in the heterogeneous cardiac muscle. The difficulties arise owing to the interaction of vortices. We investigate the excitation dynamics in the vicinity of the cores of pinned vortices. This allows us to draw conclusions about the overall dynamics. When the VW of a vortex is hit by the E-pulse, this vortex is displaced to a new position. If the vortex was situated close to the tissue boundary, it is terminated. Our aim is that VW of every vortex is hit by an E-pulse (‘all vortices are terminated’). Wave patterns were calculated using the Barkley model:

$$\begin{array}{rl} u_{t} & =\varepsilon^{-1}u(1-u)\left[u-\frac{\left(v+b\right)}{a}\right]+\nabla^{2}u,\\ v_{t} & =u-v, \end{array}$$

in a rectangular domain with circular holes, with no-flux boundary conditions at the outer boundaries.

Pulses of electric field **E** are implemented as in [26] using the boundary conditions **n**⋅(∇*u*−**E**)=0 at the boundaries of the holes. The numerical integration used an explicit Euler scheme with a time step of 1.6×10^−3^ and central-difference approximation of Laplacian with a space step of 1/6. The Barkley model is formulated in non-dimensional units; for presentation purposes, we postulate that the time unit of the Barkley model is 20 ms and the space unit of the Barkley model is 0.5 mm; this gives physiologically reasonable time and space scales.

**Figure 1.**
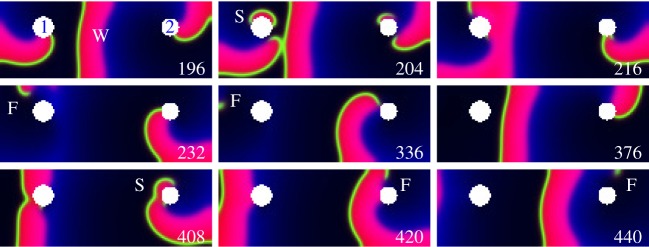
Parallel termination of two pinned vortices with geometrical locations and time positions of the critical (vulnerable) windows (VW) both unknown. The slow vortex 1 (period *T*_v1_=87 ms, pinned to the 1.2 mm defect 1, is entrained by the fast vortex 2 (period *T*_v2_=83 ms, 1.0 mm defect 2. They are paced with electric field directed from top to bottom, |**E**|=1.3 *V* *cm*^−1^, pulses 2 ms duration, period 100 ms. This induces the phase scanning with the time step *s*=17 ms. Colour code: red is a wave, green is the wavefront. Time is measured from the start of pacing at *t*=0 ms. 196 ms: a wave W emitted by vortex 2 enslaves vortex 1. 204 ms: an E-pulse delivered at *t*=200 ms induces a wave S. 216 ms: the right wavebreak of wave S annihilates with the tip of vortex 1 (they have opposite topological charges). 232 ms: vortex 1 is unpinned and terminated. The left wavebreak of S created a free vortex F. 336…376 ms: F disappears on the boundary. 408…440 ms: Next E-pulse similarly terminates vortex 2. Barkley model, parameters *a*=0.8, *b*=0.09, *ϵ*=0.02.

Figure 1 shows termination of two pinned vortices by E-pacing (see also the movie in the electronic supplementary material). This can be achieved generically, for any parameter of the vortices, without knowing their geometric location and time positions of the VWs.

**Figure 2.**
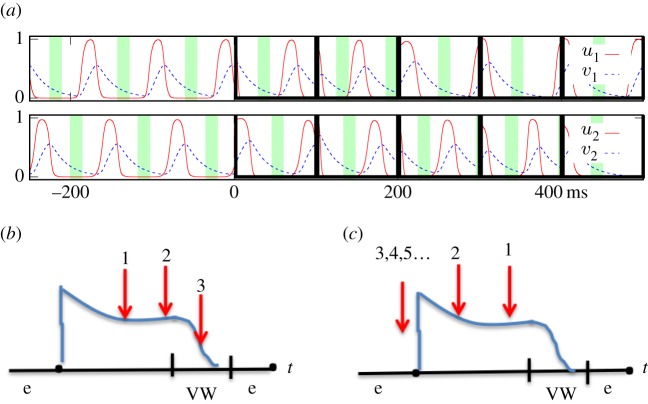
Phase scanning. (*a*) *u*_1_(*t*), *v*_1_(*t*) are recordings from the point just above defect 1, figure 1, and *u*_2_(*t*), *v*_2_(*t*) are the same for defect 2. The bold black lines indicate timing of the delivered E-pulses. Shaded areas are VWs, defined as time intervals where *v*∈(0.0871,0.18), *u*<*b*/*a*. Seen that in spite of small phase disturbances produced by E-pulses, the topological features of the scanning are not disturbed, scanning successfully terminates the vortices. E-pulse 3 (*t*=200 ms) reaches VW of vortex 1 and terminates it, compare with figure 1, *t*=204 ms, where is seen a wave S induced by an E-pulse delivered at *t*=200 ms. E-pulse 5 (*t*=400 ms) reaches VW of vortex 2 and terminates it. Compare with figure 1, t=408…440 ms: E-pulse delivered at (*t*=400 ms) terminates vortex 2. (*b*,*c*) Schematic. Superimposed action potentials (AP) are shown. Red arrows indicate timing of the delivered E-pulses; ‘e’ is an excitable gap, *s* is the scanning step, *s*=*T*−*T*_v_. (*b*) *s*>0 for *T*>*T*_v_, scanning reaches the VW. (*c*) *s*<0 for faster pacing *T*<*T*_v_, the scanning moves in the opposite direction. E-pulse reaches the excitable gap ‘e’, excites an AP thus resetting the rotation phase, and all subsequent pulses get into the same phase [25,27]. It does not reach the VW.

To hit the VW with an E-pulse, the phase scanning (figure 2*a*,*b*) should be performed with steps 0<*s*<VW. Thus, the VW duration (at the chosen **E**, see figure 4*h*) determines suitable values of *s*. Then, the number of pulses *N* required to cover the whole period of a vortex is *N*≥*T*_v_/*s*, where *T*_v_ is the period of the vortex, *s*=*T*−*T*_v_ is the scanning step, and *T* is the period of E-pacing. This gives the E-pacing period *T*=*s*+*T*_v_. Thus, all parameters of E-pacing (*E*, *N*, *T*) can be set following equations

2.1 $$0<s<\mathrm{VW}(E),\;N\geq \frac{T_{v}}{s},\;T=s+T_{v},$$

to guarantee that at least one E-pulse hits the VW.

Minimum energy for termination of a pinned vortex is achieved when the electric field strength is chosen so that the normalized $\bar{\mathrm{VW}}(E)=1/N$, where *N* is the number of pacing pulses. The maximal success rate is achieved when the pacing frequency *f*=*f*_best_, where *f*_best_ is the frequency for which the normalized scanning step $\hat{s}=1/N$. When *f*<*f*_best_, i.e. *T*>*T*_best_, the scanning step $\hat{s}>\bar{\mathrm{VW}}$, and the VW may be missed while scanning, thereby decreasing the success rate. When *f*>*f*_best_, so *T*<*T*_best_, the scanning step $\hat{s}<\bar{\mathrm{VW}}=1/N$, and not all phases are scanned. This also decreases the success rate.

What does interaction of vortices change here? In cardiac muscle, the fastest vortex entrains slower vortices if there is a normal wave propagation between them. Then, only one frequency remains; this facilitates vortices termination. But entrainment ceases if the fastest vortex is terminated before the slower vortices, and then the frequency of the system changes (period increases). Here, two wave scenarios are possible, which we describe for the case of just two vortices with periods *T*_v1_ and *T*_v2_, such that *T*_v1_>*T*_v2_.

1. If the periods of the two vortices are not much different, so that *T*_v2_<*T*_v1_<*T*, then the pacing is still under-driving, and the slower vortex (*T*_v1_) can be terminated by E-pacing with same period *T* (figures 1 and 2*b*), provided that termination conditions (2.1) are met for the slower vortex.
2. If however, the periods of the two vortices are so much different that
  2.2 $$T_{v2}<T<T_{v1},$$
  then the pacing with the same period is no longer under-driving, but over-driving. And overdrive pacing will typically entrain the remaining vortex rather than eliminate it.

For successful termination of fibrillation, the E-pacing period should be increased to a higher value *T*_2_, such that *T*_v1_<*T*_2_. Thus, vortices can be terminated in any case. Experiments [16] underestimated the potential of the method as this mechanism was not known yet.

### 2.2 Free vortices

**Figure 3.**
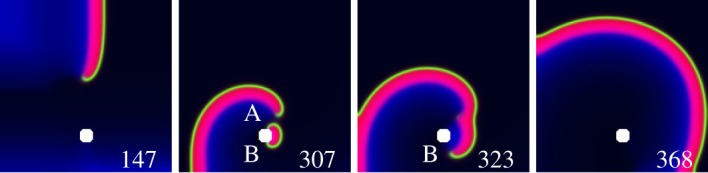
Termination of a free vortex by an E-pulse. 147 ms: a free vortex and a defect (white). 307 ms: a semicircular wave (with wavebreaks A and B) emitted from the defect by an E-pulse, electric field directed from right to left. 323 ms: wavebreak A fused with the vortex tip. 368 ms: after annihilation of wavebreak B with the border, only a wave without wavebreaks is left in the medium. Barkley model, parameters *a*=0.6, *b*=0.075, *ϵ*=0.02.

Below, we describe a mechanism terminating a high frequency free vortex by electric field pacing. Numerical and theoretical publications state it is very easy: usual local pacing (anti-tachycardia pacing; ATP) with frequency higher than a free vortex frequency terminates a free vortex. It works in experiment and in clinics, but only for low frequency vortices. Classic ATP cannot terminate ventricular fibrillation (VF), cannot terminate high frequency rotating waves, including free rotating waves. Waves emitted from a pacing electrode propagate along the whole tissue only for low frequency. For higher frequencies, the Wenckebach rhythm transformation occurs in a heterogeneous cardiac tissue. By contrast, an electric field penetrates everywhere, without frequency limitations, and only requires local heterogeneities to act as virtual electrodes. This mechanism can be used for terminating a high frequency free vortex. A free (not pinned) vortex can be terminated when its moving core passes not very far (at distance L≲λ, where *λ* is the wavelength) from a defect in the medium, serving as a virtual electrode, figure 3 (this illustration uses the same mathematical model for the excitable medium and for the action of the electrical field as in the previous subsection). The success rate increases as distance *L* decreases. A mechanism reliably terminating a free rotating wave was found in 1983 [28]: waves with a frequency higher than the frequency of a rotating wave, induce its drift and termination on the boundary. Cardiologists used a high-frequency pacing (ATP) well before the mechanism was understood. But ATP can not terminate high frequency rotating waves. This fundamental limitation is overcome by the mechanism of a free vortex termination proposed here. This mechanism depends on wave emission induced from a defect induced by the electric field. The electric field penetrates everywhere, hence no restriction on its efficacy is imposed by the maximal frequency of propagating waves in any part of the cardiac tissue.

**Figure 4.**
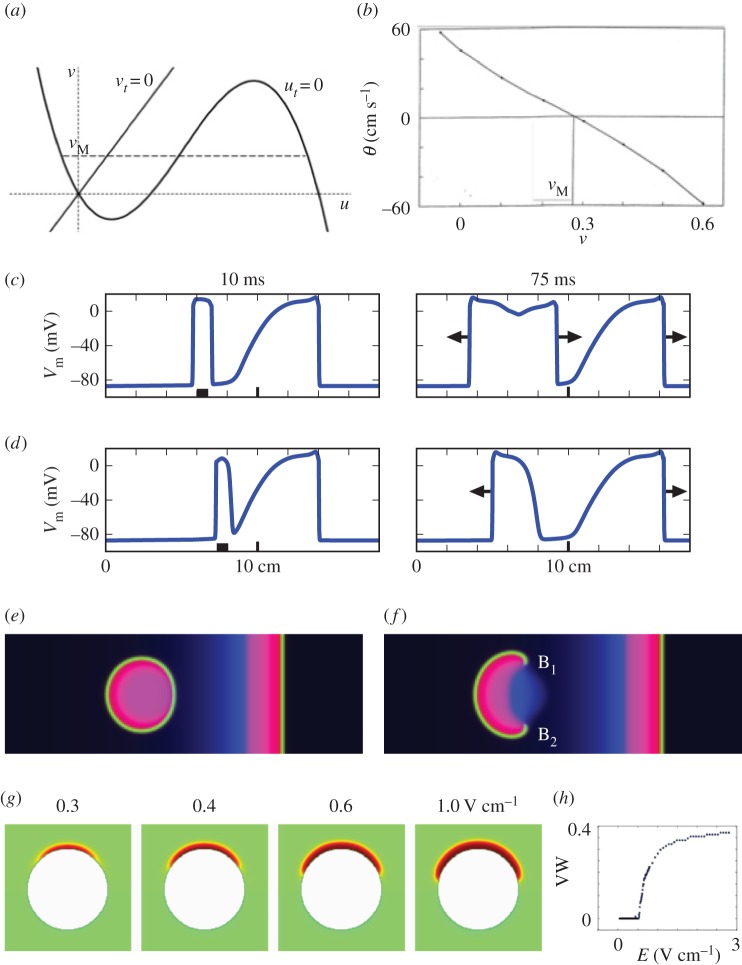
Change of topological charge, creation of phase singularities and vulnerable window (VW). (*a*–*d*) *One-dimensional mechanism*. (*a*) Nullclines of FHN equations. *M* is the Maxwell point. The topological charge of a wave pattern is changed by an E-pulse only when an image of a nucleated wave contains the Maxwell point. (*b*) Wave front velocity *θ* versus the slow variable *v*. The value of *v* corresponding to velocity *θ*=0 is the ordinate *v*_*M*_ of the Maxwell point on panel (*a*). (*c*) The topological charge conservation in one dimension. Generic case: an electric pulse 3 ms in duration is delivered far from the tail of an action potential (AP). *t*=10 ms after pacing: a nucleated wave, very narrow, and the electrode (black square) below it. *t*=75 ms: the nucleated wave developed into two counter propagating APs. Their total topological charge is zero. (*d*) Violation of the topological charge conservation. *t*=10 ms: an electric pulse is delivered closer to the tail of the AP, inside VW. *t*=75 ms: only one AP is induced. It propagates to the left only. The topological charge is changed. Cardiac ionic model by Majahan *et al.* [29]. (*e*,*f*) *two-dimensional mechanism*. (*e*) No phase singularities are created. An electric pulse is delivered as in (*c*). (*f*) Creation of two phase singularities, B1 and B2. An electric pulse is delivered as in (*d*). (*g*,*h*) VW increases with electric field in two dimensions. (*g*) Mechanism: the larger *E*, the larger is the depolarized region. (*h*) Graph *VW*(*E*).

An increased amplitude of electric field |**E**| results in defibrillation. There is a classical explanation: electric field should be increased to the value where it terminates all propagating waves. A physical explanation is: the wave emission is induced from a larger number of defects when the electric field is increased [14]. Figure 4*g* shows another mechanism: the size of the excited region increases with the electric field, and the duration of the VW increases with it.

### 2.3 Time separation analysis of vulnerable window

The mechanism of the VW is related to the change of topological charge in one dimension and the creation of new topological singularities in two dimensions.

The topological charge (or ‘winding number’, in terminology of [8]) in a one-dimensional closed circuit can be defined as the increment of the excitation phase per one loop around the circuit, where the excitation phase can be defined as the angle in the polar coordinates in the phase plane of the reaction kinetics, centred at a suitably chosen point in the ‘No Mans Land’, in terminology of [30]. Likewise, topological singularity in two dimensions is defined as a point such that any sufficiently small one-dimensional contour surrounding it has a topological charge. We illustrate the relationship between these concepts and VW using time-separation analysis for the FitzHugh–Nagumo (FHN) equations:

2.3 $$u_{t}=f(u)-v+Du_{xx}$$

and

2.4 $$v_{t}=\varepsilon (u-kv).$$

Here *f*(*u*)=*Au*(1−*u*)(*u*−*α*), and *ϵ*≪1 is a small parameter permitting the time-scales separation (for details of relevant formalisms, see review [31]). The wavefront propagation velocity *θ* can be estimated by assuming that the slow variable *v* is approximately constant across the wavefront. The propagation of the front is then described by equation (2.3) alone, where *v* is a constant parameter. Transforming the independent variables such that *ξ*=*x*−*θt* makes equation (2.3) an ordinary differential equation:

$$-\theta u_{\xi }=f(u)-v+Du_{\xi \xi },$$

which together with boundary conditions $u(\infty )=u_{1}$, $u(-\infty )=u_{3}$, where *u*_1_=*u*_1_(*v*) and *u*_3_=*u*_3_(*v*) are, respectively, the lowest and highest roots of *f*(*u*)=*v*, define *θ* as a function of *v* (figure 4*b*). Here, velocity *θ*(*v*) is negative for *v*>*v*_*M*_, where *v*_*M*_ is the Maxwell point, $\int_{u_{1}(v_{M})}^{u_{3}(v_{M})}(f(u)-v_{M})\;du=0$, *θ*(*v*_*M*_)=0 [32].

Vulnerability is a cardiological term coined for initiation of fibrillation by an electric pulse. In the physical language, vulnerability in one dimension can be related to a change of the topological charge, and in two dimensions and three dimensions to creation of new phase singularites. In one dimension, this phenomenon happens when the current injection nucleates a wave propagating in only one direction, figure 4*d*. This is in contrast with the generic case, where the topological charge is conserved, when the new wave propagates in two directions, figure 4*c*, or new wave is not nucleated at all (not shown). For one-directional propagation to happen, the nucleated wave should cover the points which have *v*=*v*_*M*_ corresponding to the Maxwell point *θ*=0. Then, a part of the nucleated wave has positive velocity (becoming the front of the wave) and another part has a negative velocity (becoming the tail of the wave), as in figure 4*d*,*f*. Otherwise, all parts of the nucleated wave have velocity of the same sign. When velocity *θ*<0, the nucleated wave shrinks and decays. In the opposite case, it enlarges in all directions, as in figure 4*c*,*e*.

### 2.4 Axiomatic model based on properties of vulnerable window

Now, we formulate an axiomatic model based on the properties of VW discussed above. Let $\phi_{n}^{j}\in [0,1)$, *j*=1,2, *n*=1,…,5 describe the phase of *j*th vortex just after the delivery of the *n*-th E-pulse, *T*_*j*_ be the natural periods of the vortices, *T*_2_>*T*_1_, and correspondingly ${\hat{s}}_{j}=\hat{T}-{\hat{T}}_{j}=(T-T_{j})/T_{d}$ are the scanning steps normalized by the measured dominant period, *T*_*d*_. We postulate $\phi_{n+1}^{j}=(\phi_{n}^{j}+s)\;\mathrm{mod}\;\;1$, subject to the following corrections: (i) if $\phi_{n+1}^{j}\in [1-\bar{\mathrm{EG}},1)$, where $\bar{\mathrm{EG}}$ is the normalized duration of the excitable gap, then $\phi_{n+1}^{j}$ is replaced with 0: this describes resetting the *j*th phase by the E-pulse; (ii) if $\phi_{n+1}^{j}\in [1-\bar{\mathrm{EG}}-\bar{\mathrm{VW}},1-\bar{\mathrm{EG}})$, where $\bar{\mathrm{VW}}$ is the normalized duration of the VW, then the *j*th vortex is considered terminated; (iii) if neither vortex is terminated, then the slower vortice's phase is enslaved by the faster one's, $\phi_{n+1}^{2}=(\phi_{n+1}^{1}-D)\;\mathrm{mod}\;\;1$, where *D* is a fixed phase delay; and (iv) if both vortices are terminated, iterations stop and E-pacing is deemed successful.

**Figure 5.**
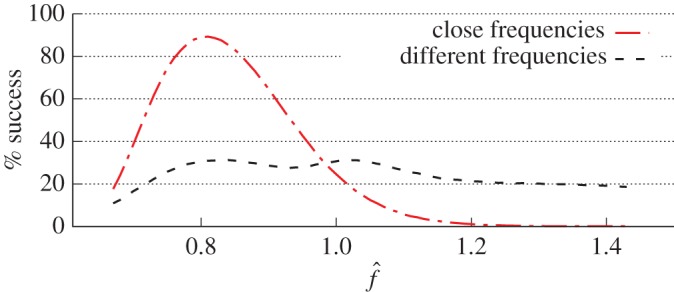
Success rate of two vortices termination. Success rate versus normalized frequency $\hat{f}=f/f_{d}$ where *f*_*d*_ is the dominant frequency. On the image, ‘different’ and ‘close’ frequencies mean significant and insignificant difference in frequencies as per equation (2.2). Numerical calculations with the normalized $\bar{\mathrm{VW}}$ =0.2.

Figure 5 shows the success rate of termination of two vortices as a function of the normalized frequency of E-pacing. The graphs represent results of Monte Carlo simulations of the axiomatic model described above, with random initial phases of vortices and two variants for the choice of frequencies: (i) normal distributions of parameters ${\hat{T}}_{1}=1\pm 0.1$ and ${\hat{T}}_{2}=1.6\pm 0.05$ (mean ± s.d.), ‘different frequencies’; and (ii) the same parameters for ${\hat{T}}_{1}$, and ${\hat{T}}_{2}$ enforced very close to ${\hat{T}}_{1}$, namely ${\hat{T}}_{2}=(1+10^{-6}){\hat{T}}_{1}$, ‘close frequencies’, with other parameters fixed at $\bar{\mathrm{EG}}=0.4$, $\bar{\mathrm{VW}}=0.2$, *D*=0.25. The success rate of termination of two vortices with significant difference in frequencies as per equation (2.2) is seen in figure 5 to be threefold lower than that for vortices with insignificant difference in frequencies. This happens because when the leading (fastest) vortex is terminated first, the same E-pacing period *T* appears below the period *T*_1_ of the resting slower vortex, see equation (2.2). Thus, the resting vortex cannot be terminated (figure 2*c*).

Vortices termination can be induced also by other mechanisms different from vulnerability, e.g. pacing-induced drift of a free vortex [28], unpinning of weakly pinned vortices [33,34] and by three-dimensional mechanisms [35–37].

## 3. Experiment

**Figure 6.**
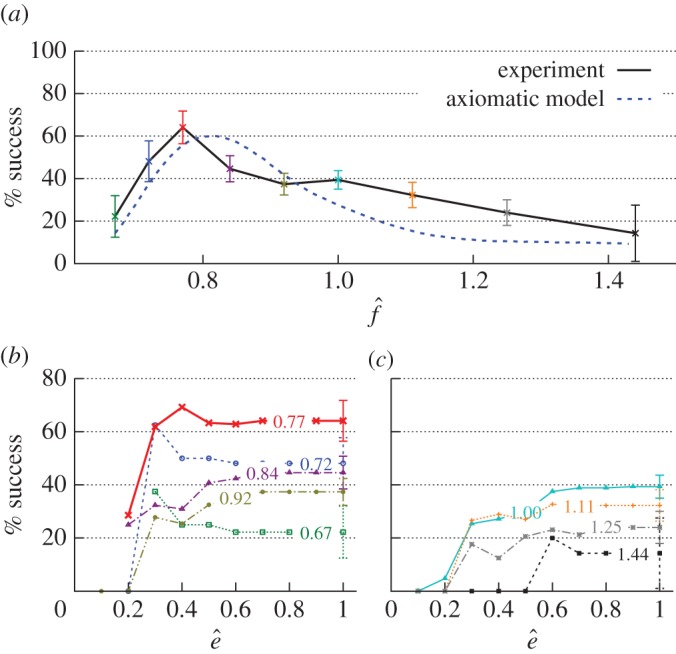
Fibrillation termination in the isolated pig hearts. The success rate of defibrillation in 486 experiments by five biphasic E-pulses. (*a*) Success rate versus normalized frequency $\hat{f}=f/f_{d}$ where *f*_*d*_ is the dominant frequency of fibrillation. Error bars: the standard deviation. The blue curve is obtained by mixture (50 : 50) of two theoretical curves shown in figure 5. (*b*,*c*) Success rate for defibrillation energies not exceeding $\hat{e}$, for frequencies $\hat{f}$ shown near each curve. Normalized energy $\hat{e}=e/e_{1}$, where *e*_1_ is the threshold *E*_50_ energy of defibrillation by 1 shock. (*b*) shows graphs for $\hat{f}<1$, (*c*) shows graphs for $\hat{f}\geq 1$. Graphs (*b*,*c*) and the experimental curve in (*a*) are calculated from data in [38]. Image (*a*) indicates that in about a half of fibrillation experiments, the frequencies of the vortices were not significantly different. The optimal pacing frequency $\hat{f}=0.77$ is below the arrhythmia frequency $\left(\hat{f}<1\right)$ as it should be for terminating pinned vortices. These experiments provide evidence that pinned vortices, hidden from direct observation, are significant in fibrillation.

Results of about 500 experiments with vortices termination in the isolated pig hearts are presented in figure 6. Fibrillation was induced and terminated as in [15,16]. In terms of the normalized pacing frequency $\hat{f}$, the numbers *n* of the experiments were: *n*=18 for $\hat{f}=0.67$; *n*=28 for $\hat{f}=0.72$; *n*=39 for $\hat{f}=0.77$; *n*=65 for $\hat{f}=0.84$; *n*=91 for $\hat{f}=0.92$; *n*=127 for $\hat{f}=1$; *n*=62 for $\hat{f}=1.11$; *n*=50 for $\hat{f}=1.25$ and *n*=7 for $\hat{f}=1.44$.

In figure 6*a*, the experimental curve is fit by the blue theoretical curve much better than by either of the two theoretical curves in figure 5. It indicates that in about a half of fibrillation experiments, the frequencies of the vortices were not significantly different.

Figure 6*a*,*b* shows that the optimal pacing frequency $\hat{f}=0.77$ is below the arrhythmia frequency $\left(\hat{f}<1\right)$ as it should be for terminating pinned vortices. Note that elimination of a free, rather than pinned, vortex by inducing its drift via the mechanism described in [28], requires the pacing frequency to be above the arrhythmia frequency, $\hat{f}>1$. These experiments provide evidence that pinned vortices, hidden from direct observation, are significant in fibrillation. In particular, they show that the VW mechanism is an explanation for the high success rate of VF termination using electric field pacing.

## 4. Discussion

In this paper, after more than 25 years of research, we propose a solution to a problem; how to terminate multiple vortices in the cardiac tissue hidden from direct observation. In order to control vortices, two problems should be overcome: both, the geometric positions of their cores, and the positions of their critical time windows, are not known during fibrillation. The first problem we have solved previously using an electric field pulse to excite the cores of all pinned vortices simultaneously. Approaches to solve the second problem are being developed. One of them is based on the phase scanning of all pinned vortices in parallel to hit the critical time window of every pinned vortex. In this paper, we investigate the related physical mechanisms using simple two variable models as well as a detailed ionic model of the cardiac tissue. A similar mechanism terminates also a free (not pinned) vortex, when the vortex's core passes not very far from a defect.

Even though it is widely believed that the success of defibrillation has a probabilistic nature, we have shown that termination of one vortex can be achieved deterministically, in any case. This can be achieved generically, for any parameters of the vortex, without knowing its geometric location and timing of its VW. All that is needed is to set the parameters of E-pacing (*E*,*N*,*T*) according to equations (2.1). Termination of an arrhythmia becomes probabilistic when two or more vortices are involved. If there is normal wave propagation between the two vortices, and the slower vortex is enslaved by the faster one, then the E-pacing protocol described in [16] cannot control which of the vortices will be terminated first. If the slower vortex is terminated first, the frequency of the system does not change, and both vortices are terminated deterministically, in any case. If, by chance, the faster vortex is terminated first, the frequency of the system changes, and the remaining slower vortex may be not terminated if conditions (2.2) are satisfied.

Here, we investigated two extreme cases: permanently pinned vortices and permanently free vortices. There is no sharp transition between them in heterogeneous media with different size pinning centres. In cardiac muscle, there are heterogeneities of all sizes, including those to which vortices pin weakly. A weakly pinned vortex is pinned for some time only, then leaves the pinning centre and moves as a free vortex, again for some time. When moving and meeting a pinning centre, it may pin to it, or may reach the boundary of the tissue and disappear.

Three-dimensional models are widely used in investigation of wave patterns induced by rotating waves, e.g. [10]. A three-dimensional mechanism of defibrillation was described in [35–37]. Study of vortices termination in two-dimensional models is a necessary step for developing an understanding of mechanisms of three-dimensional vortices termination in the heart. Termination of vortices underlying fibrillation is only a small part of a problem preventing and curing the cardiac arrhythmias where a combination of molecular and dynamics approaches is prominent [39].

In conclusion, we have shown mechanisms of terminating pinned and free vortices by electric field pulses when the geometric positions of their cores, and the phases of rotation are not known. These results form the physical basis for creation of new effective methods for terminating vortices underlying fibrillation.

## Data accessibility

A movie version of figure 1 is available as the electronic supplementary material.
